# Child Killed by a Chicken

**Published:** 1875-08

**Authors:** 


Child Killed by a Chicken.—We find in the Rome *Daily Commercial* a notice of this extraordinary occurrence in the case of an infant child of Mr. A. J. Langley, living near Gadsden, Ala. The child, eighteen months old, whilst at play in the yard, was furiously attacked by a rooster, knocked down and spurred several times. Dr. Ewing, who attended, states that one stroke of the spur had entered the brain through an open suture of the skull, and resulted in death to the child. The doctor thinks this the first case of the kind in the history of the world.

